# What influences quality of life in older people living with HIV?

**DOI:** 10.1186/s12981-017-0148-9

**Published:** 2017-04-11

**Authors:** Jose Catalan, Veronica Tuffrey, Damien Ridge, Dana Rosenfeld, On behalf of the HALL (HIV and Later Life) Team

**Affiliations:** 1 CNWL NHS Foundation Trust, South Kensington and Chelsea Mental Health Centre, London, UK; 2 Faculty of Science and Technology, University of Westminster, London, UK; 3 School of Social Science and Public Policy, Keele University, London, UK

## Abstract

**Background:**

People with HIV with access to treatment are growing older and living healthier lives than in the past, and while health improvements and increased survival rates are welcome, the psychological and social consequences and quality of life of ageing are complex for this group. Understanding how ageing, HIV and quality of life intersect is key to developing effective interventions to improve QoL.

**Methods:**

One hundred people with HIV over the age of 50 (range 50–87, mean 58), were recruited through HIV community organizations, and clinics, and included men who have sex with men (MSM), and Black African and White heterosexual men and women. The WHOQOL-HIV BREF was used, as well as the Every Day Memory Questionnaire, and additional questions on anxiety and depression to supplement the WHOQOL.

**Results:**

While most rated their quality of life (QoL) positively, bivariate analysis showed that better QoL (total score and most domains) was strongly associated with being a man; in a relationship; in paid employment; having higher level of income; not on benefits, and to a lesser degree with being MSM, having higher level of education, and diagnosed after the age of 40. Multivariate analysis showed that not being on benefits was the variable most consistently associated with better quality of life, as was being partnered. Concerns about everyday memory difficulties, and anxiety and depression scores were strong predictors of poorer quality of life.

**Conclusion:**

While the cross-sectional nature of the investigation could not establish that the associations were causal, the findings indicate that concerns about memory difficulties, anxiety and depression, as well as gender, ethnicity, financial factors, and relationship status, are important contributors to QoL in this group. These findings point towards the need for further research to clarify the mechanisms through which the factors identified here affect QoL, and to identify possible interventions to improve the QoL of older people living with HIV.

## Background

People living with HIV with access to effective antiviral treatments nowadays are growing older and living healthier lives than they were before these treatments became available in the mid-1990s [1, 2], while more people are acquiring HIV at an older age [3, 4]. In the UK there are now more than 25,000 people aged 50 and older living with HIV [4]. Yet, while health improvements and much increased survival rates are to be welcomed, the psychological and social consequences of ageing with HIV are complex. Although the individual impact of getting older with HIV can be positive e.g. [5, 6], the problems faced by older people with HIV include high levels of stigma [7–10]; concerns about dependence on benefits [11], ongoing concerns about disclosure [12], and uncertainty over how ageing, HIV, and its treatment affect health [13]. Gender, ethnicity, sexuality, and social supports play an important role in how older people living with HIV adapt [5, 14–18]. There is evidence for poorer reported quality of life (QoL) in older people living with HIV compared with younger people with the infection [19–21], although this has not been a consistent finding [22, 23]. None of these studies has included a control group without HIV. There is concern about the physical impact of HIV infection in older people, both in terms of non-AIDS related morbidity [24], and cognitive function [25–27]. Understanding how ageing, HIV, and QoL intersect is key to furthering our knowledge and to developing appropriate interventions to improve their QoL.

## Aims

This paper is based on analysis of quantitative data gathered for the multi-method HIV and Later Life (HALL) Study [18]. The aims of the study were to uncover self-reported QoL, social supports, and mental health among older people living with HIV in the UK; identify links between them; and identify factors associated with reported mental health and QoL in this population. The study received ethical approval from Keele University and from the NHS-Research Ethics Committee in London.

## Methods

### Participants

A total of 100 (73 men and 27 women) people over the age of 50 years living with HIV, including 76 interviewees and 24 on line participants, completed the self-administered questionnaires. In terms of ethnicity, 57 were White British, 13 White other, 28 Black African, and 2 Black other. There were 51 MSM, 45 heterosexual, and 4 bisexual participants. Age ranged from 50 to 87 years, median 56 (see Rosenfeld et al. [18] for further details).

Participants were recruited through London-based HIV community organizations and HIV clinics. Staff and volunteers reviewed records to identify patients who met the study inclusion criteria, including having being diagnosed with HIV for at least 12 months, and not suffering acute or severe psychological symptoms. Surveys were completed immediately post-interview, and 24 surveys were completed on-line by non-interviewed participants. There were no differences between participants that completed the surveys post-interview and online in terms of their QoL scores, memory concerns, anxiety, depression, proportions partnered, educational level, on benefits, paid employment, living alone, attending support groups, and time since diagnosis.

### Instruments

The WHOQOL- HIV BREF [28], a self-reported 31-item questionnaire, was used. It gives a multidimensional profile across six domains (physical health; psychological health; level of independence; social relationships; environment; and spirituality), as well as an overall rating of QoL.

The Every Day Memory Questionnaire (EMQ), a self-reported 13-item questionnaire was used to establish the subjective perception of cognitive failure [29].

Participants were asked to rate their anxiety and depression on a scale from 0 (not at all) to 10 (extremely) from the Bournemouth Questionnaire, a self-report instrument that has been used in other populations with medical disorders [30].

### Analysis

Data were analysed using the statistical package for Social Science Version 18.0 (SPSS, Chicago, Illinois, USA). The independent samples *t* test was used to compare scores for each QoL domain, total QoL, and memory by binary categories of exposure variables. Since the anxiety and depression ratings were derived from single questions, and the shape of their distributions was not normal, a non-parametric test (Mann–Whitney) was used to compare scores by binary categories of exposure variables instead of *t* tests. Two-tailed tests with p < 0.05 were considered statistically significant. p values were not adjusted for multiple testing because the objective was simply to identify patterns of associations.

Stepwise linear regression was used to identify the best combination of exposure variable to predict the outcomes of interest. The process repeatedly applies multiple regression, each time searching for the predictor that has the highest correlation with the outcome variable, and removing the weakest correlated variable (the criterion for entry was p < 0.05 and for removal p > 0.10). The final model includes the variables that best explain the distribution.

It was expected that multicollinearity (correlation between predictor variables) could affect the parameters of the regression models, and lead to poor estimation of the regression coefficients. The variance inflation factor was used to assess whether correlation between independent variables in the multivariate models was of concern.

## Results

### Response to single questions on overall QoL, anxiety and depression in the last 4 weeks

Over half of the participants (56%) rated their QoL as ‘good or very good’, 24% as ‘neither good nor poor’, 9% as ‘poor’, and 1% as ‘very poor’.

For the anxiety and depression scores, on a scale of 1–10 (where 0 is not at all anxious or depressed, and 10 is extremely anxious or depressed), the median scores for both variables were 5, with 26% reporting 0 or 1 for depression, and 22% reporting 0 or 1 for anxiety. For both variables 12% of the participants scored the highest scores of 9 or 10.

### Bivariate analysis of WHOQOL-HIV BREF scores versus demographic, socio-economic, social support, and year of diagnosis

Table 1 shows the findings from the bivariate analysis of QoL. Men reported higher QoL, total QoL score (p < 0.01) and in several domains: physical (p < 0.01), level of independence (p < 0.01), environment (p < 0.01), and social relationships (p < 0.05). White participants reported higher scores on the environment domain only (p < 0.01). Partnered participants reported a higher total QoL score (p < 0.01), and for the social relations (p < 0.01), psychological, environment, and level of independence domains (all p < 0.05). MSM reported higher scores for environment (p < 0.01), and social relations (p < 0.05).

**Table 1:** Findings from bivariate analysis of quality of life and memory scores v. binary demographic, socio-economic and social support variables (one-way ANOVA)

		WHOQoL-HIV domain scores						Total WHOQoL-HIV score	Everyday memory score	Trends
		Physical health	Psychological health	Independence	Social relations	Environmental health	Spirituality			
Mean (SD)		13.0 (3.8)	12.8 (3.3)	12.9 (3.9)	12.9 (3.4)	14.4 (2.9)	13.7 (3.3)	79.7 (16.6)	1.4 (1.1)	
Gender	*p*	0.008**	0.133	0.001**	0.038*	0.002**	0.052	0.002**	0.137	Men (27) have higher QoL values and lower memory scores than women (73)
Ethnicity	*p*	0.774	0.512	0.051	0.568	0.004**	0.581	0.382	0.481	White group (70) have higher QoL values and lower memory scores than Black group (30)
Partnership	*p*	0.129	0.036*	0.031*	0.001**	0.038*	0.119	0.009**	0.248	Partnered group (33) have higher QoL values and lower memory scores than singles group (67)
Sexual orientation	*p*	0.315	0.496	0.247	0.033*	0.001**	0.723	0.086	0.717	MSM (46) have higher QoL values and lower memory scores than Other group (53)
Age	*p*	0.152	0.340	0.167	0.993	0.242	0.417	0.230	0.386	Those aged 50–55 years (55) have higher QoL values and lower memory scores than those aged ≥56 years (45)
Education	*p*	0.024*	0.471	0.021*	0.722	0.385	0.905	0.151	<0.001***	Those with higher education (57) have higher QoL and lower memory scores than those without HE (43)
Income	*p*	0.004**	0.236	<0.001***	0.040*	<0.001***	0.208	0.001**	0.001**	Those earning ≥£10 K/year (45) have higher QoL and lower memory scores than those earning <£10 K/year (42)
Benefits	*p*	<0.001***	<0.001***	<0.001***	0.002**	<0.001***	0.026*	<0.001***	<0.001***	Those not on benefits (45) have higher QoL values and lower memory scores than those receiving benefits (55)
Children	*p*	0.461	0.759	0.151	0.150	0.073	0.565	0.294	0.522	Those without children (48) have higher QoL values and lower memory scores than those with children (52)
Grand-children	*p*	0.116	0.180	0.001**	0.066	0.001**	0.316	0.012*	0.051	Those without grandchildren (65) have higher QoL and lower memory scores than those with grandchildren (24)
Working	*p*	0.004**	0.019*	<0.001***	0.004**	<0.001***	0.026*	<0.001***	<0.001***	Those in paid work (28) have higher QoL values and lower memory scores than those with no paid work (72)
Live alone	*p*	0.669	0.593	0.797	0.817	0.971	0.342	0.922	0.748	Inconsistent direction of difference between those living alone (59) and those living with someone (41)
Religious	*p*	0.793	0.483	0.076	0.102	0.001**	0.738	0.113	0.102	Those who rarely or never attend services (52) have higher QoL and lower memory scores than those who attend (48)
Support group	*p*	0.874	0.164	0.291	0.807	0.074	0.490	0.824	0.603	Inconsistent direction of difference between those who do not attend a support group (45) and those who attend (55)
Year of diagnosis	*p*	0.149	0.219	0.109	0.427	0.727	0.411	0.184	0.653	Those diagnosed during or after 1997 (77) have higher QoL and similar memory scores to those diagnosed earlier (21)
Age of diagnosis	*p*	0.030*	0.019*	0.021*	0.213	0.285	0.267	0.031*	0.168	Those diagnosed at age 40 years or younger (23) have lower QoL values and higher memory scores than those diagnosed after the age of 40 (75)

Findings from one-way ANOVA * 0.05 > *p* ≥ 0.01 ** 0.01 > *p* ≥ 0.001 *** p < 0.001

Participants diagnosed with HIV at 40 or younger had worse QoL than those diagnosed with HIV over the age of 40, with worse total QoL score (p < 0.05), and worse physical (p < 0.05), psychological health (p < 0.05), and level of independence (p < 0.05) scores.

Those with higher education reported higher QoL in physical and level of independence (both p < 0.05). Participants earning more than £10,000/year reported higher total QoL score (p < 0.01), and scored higher for level of independence and environment (both p < 0.001), physical (p < 0.01), and social relations (p < 0.05). Those not receiving state benefits had a higher QoL in total score (p < 0.001), and in all domains: physical, psychological, level of independence, and environment (all p < 0.001), social relations (p < 0.01) and spiritual (p < 0.05). Participants in employment reported higher QoL in total score (p < 0.001), and in all domains: level of independence and environment (both p < 0.001), physical, and social relations (both p < 0.01), and psychological and spiritual (p < 0.05).

Participants without grandchildren reported better QoL with respect to total score (p < 0.05), and to level of independence and environment (both p < 0.01). Those who attended religious services had higher QoL for the environment domain (p < 0.01).

### Multivariate analysis of WHOQOL scores

Stepwise regression was used to identify the best combination of exposure variables to predict the QoL scores. Table 2 shows the findings from stepwise multiple regression analysis of QoL scores by binary demographic, socio-economic and social support variables. Exposure variables with p < 0.05 in the bivariate analysis reported above were included for potential selection in the regression models. Findings from the exposure variables gender, income, age of diagnosis, and religious attendance are not included in the table, as these variables were no longer significant when included in models together with the other variables. The findings for those variables finally selected by the stepwise process for inclusion in each regression model are shown in the table (the criterion for entry was p < 0.05, and for removal was p > 0.10).

**Table 2:** Findings from multivariate analysis of WHOQoL-HIV quality of life and memory scores v. binary demographic, socio-economic and social support variables

	WHOQoL-HIV domain scores												Total WHOQoL-HIV score		Everyday memory score^a^	
	Physical health		Psychological health		Level of independence		Social relations		Environmental health		Spirituality					
	Coeff.	*p*	Coeff.	*p*	Coeff.	*p*	Coeff.	*p*	Coeff.	*p*	Coeff.	*p*	Coeff.	*p*	Coeff.	*p*
Constant	17.83	<0.001***	15.68	<0.001***	14.65	<0.001***	11.68	<0.001***	15.23	<0.001***	16.77	<0.001***	86.54	<0.001***	0.80	0.004**
Ethnicity (White)	−3.25	0.002**	−1.65	0.044*							−1.83	0.024*			0.45	0.046*
Partnership (partnered)					1.78	0.029*	2.28	0.008**					8.32	0.027*		
Sexual orientation (MSM)									1.47	0.012*						
Education (with higher education)															−0.47	0.023*
Benefits (on benefits)	−3.38	<0.001***	−2.62	0.001*	−3.73	<0.001***			−2.48	<0.001***	−2.63	0.001**	−14.54	<0.001***	0.74	0.001**
Grand-children (grandparent)	−2.10	0.048*														
Working (paid work)							2.00	0.018*								
Adjusted R^2^	0.23	<0.001***	0.14	0.002**	0.33	<0.001***	0.16	0.001**	0.29	<0.001***	0.15	0.001**	0.27	<0.001***	0.22	<0.001***
N	89^b^		100		100		100		99		100		100		100	

* 0.05 > *p* ≥ 0.01 ** 0.01 > *p* ≥ 0.001 *** p < 0.001

Exposures with p < 0.05 in the bivariate analysis (Table 1) were included for potential selection in regression models, applying the stepwise linear regression process. The table shows significance values and unstandardized coefficients for those variables selected for inclusion in models, having applied criteria p to enter <0.05 and p to remove <0.10

^a^Higher scores for everyday memory score indicate greater memory difficulties, so the coefficients for memory have opposite signs to those for QoL scores

^b^The sample size included in the analysis is lower for this outcome variable because only 89 of the 100 participants responded to the question about being grandparents

Existence of problematic levels of correlation between the independent variables in the multivariate models was checked using the variance inflation factor (VIF) using a threshold of five to indicate high correlation. In the regression models the highest value of VIF was 3.1, and most values were below two, so multicollinearity is unlikely to have adversely affected selection of the best predictors.

The exposure variable benefits remained a strong predictor of total QoL and all QoL domain scores (except social relations) in the final regression models. For the social relations domain, the best combination of predictors was partnership (p < 0.01) and paid employment (p < 0.05).

After adjusting for the exposure variable of benefits, Black African ethnicity was associated with better QoL in three domains: physical (p < 0.01), psychological (p < 0.01), and spiritual (p < 0.05), in contrast with the bivariate analysis, that showed no differences between ethnic groups in QoL (apart from the environmental domain for which those of white ethnicity had significantly higher scores, p = 0.004). The significant association between sexual orientation and QoL was still evident for the environment domain (p < 0.05) after adjustment for the exposure variable benefits, but this association did not remain evident for the social relations domain. After adjusting for being on benefits, being partnered was still associated with better scores for total QoL (p < 0.05), and for the domains of social relations (p < 0.01) and independence (p < 0.05).

The exposure variable grandchildren was a significant predictor of QoL on the physical health domain after adjustment for being on benefits, whereby higher QoL scores were observed in those who were not grandparents.

### Anxiety, depression, every day memory difficulties, and WHOQOL

Findings from the analysis of the associations between the scores derived from single questions on anxiety and depression, and the binary variables, are similar to the findings from the analysis of the QoL scores for the psychological health domain (results in Table 1), apart from the finding that both anxiety and depression are positively associated with being in the low income group (both p < 0.05 with the Mann–Whitney test).

Total score for QoL was highly associated (negatively) with depression score (Spearman’s rho −0.62, p < 0.001) and anxiety score (Spearman’s rho −0.58, p < 0.001): the better the QoL, the lower the levels of reported anxiety and depression. Total score for QoL was also strongly negatively correlated with everyday memory score (Spearman’s rho −0.61, p < 0.001) (higher scores for this variable indicated more memory difficulties).

In the bivariate analysis, lower everyday memory scores were associated with higher education (p < 0.001), and with higher income (p = 0.001), not being on benefits (p < 0.001), and with being in paid employment (p < 0.001) (see Table 1). However, multivariate analysis, adjusting for exposure to other variables, showed a change in the direction of the association between everyday memory scores and ethnicity, so that participants of Black African ethnicity reported lower memory scores (indicating fewer memory difficulties).

A key finding was the association between reported memory difficulties and QoL. When the variable everyday memory score was included as a potential predictor variable in stepwise regression analysis, together with the same demographic, socio-economic, and social support exposure variables that had been included in the analysis reported above, score on the memory difficulties questionnaire was the strongest predictor of total QoL score (p < 0.001). Again, statistical criterion for entry was p < 0.05, and for removal was p > 0.10. The best combination of exposure variables to predict the total QoL score were Everyday Memory Questionnaire score (negative effect p < 0.001); being on benefits (negative effect p < 0.01); being partnered (positive effect p < 0.05); and having higher education (negative effect p < 0.05). The unexpected direction of effect of higher education is due to having adjusted for the very high correlation between higher education and memory score (see Table 1). This residual negative correlation of higher education with QoL suggests that in addition to the strong positive effect of higher education on QoL via improved cognitive functioning, there may exist an additional weak adverse effect, and while it may be spurious, this finding should be borne in mind for future investigations.

## Discussion

Most study participants reported their QoL to be ‘good’ or ‘very good’ and reported low levels of depressive or anxious self-ratings. Bivariate analysis showed that better QoL was strongly associated with male gender, being in a relationship, in employment and not on state benefits, and having higher level of income; and to a lesser extent with being MSM, having higher education, and having being diagnosed after the age of 40. Multivariate analysis demonstrated that not being on state benefits was most consistently associated with better quality of life, as was being partnered. Concerns about everyday memory difficulties, and anxiety and depression scores were strong predictors of poor quality of life.

These results were comparable to those of Owen and Catalan [5] and Ribeiro Nobre and colleagues [6], whose participants, all older people living with HIV, reported finding meaning in life and having a positive attitude towards the future. In contrast, in a study comparing younger and older people living with HIV and using the same QoL instrument used in our study, Monteiro and colleagues [21] found older people living with HIV to have lower QoL in a number of domains (physical, level of independence, and social relationships), as well as more depressive symptoms. Differences in the heterogeneity of the populations and transmission categories in the studies, and the use of different instruments to measure depressive symptoms, as well as differences in the medical and social context of the participants may explain the discrepancies between these two studies.

Interestingly, two other studies using the same instrument to measure QoL as Monteiro and colleagues [21] and ourselves also gave contrasting results, Skevington and colleagues [23] not finding older people with HIV to have poorer quality of life than younger ones, while the WHOQoL HIV group [19] found older people to have worse quality of life than younger ones. The use of the same instrument suggests that the discrepant results are related to sampling and population differences: the former study [23] carried out their investigation in samples from 23 areas, including developing and developed ones, while the latter [19] included samples from six areas, most of them developing countries. Socio-economic factors and the context of medical services are likely have influenced the participants’ reported quality of life. These inconsistent findings in relation to age and QoL in the population of people living and ageing with HIV requires further research.

Reported memory difficulties, and depressive and anxious feelings, were strongly associated with lower QoL. Although neurocognitive disorders in older people with HIV are more common than in younger people [25–27], subjective reports of memory problems do not always indicate the presence of cognitive impairment. Rather, these problems may reflect wider concerns over the ways in which age and HIV intersect to shape physical and mental health, which our analysis of qualitative data also revealed [13], and it is important to ensure appropriate cognitive screening [31].

Better economic indicators were shown to be strongly associated with better QoL, as reported by Monteiro and colleagues [21]. While not surprising, it highlights the risks that as people living with HIV continue ageing, financial independence is frequently further compromised [11].

Male gender was a strong predictor of better QoL, in contrast with the findings of the WHOQOL-HIV field test (WHOQOL-HIV Group, 2004). In agreement with our findings, Miners and colleagues [32] in a large study with a general population control group also found male gender to be associated with better quality of life. Male participants in our study were mostly MSM, and thus belonged to a group with a relatively lower intra-group stigmatisation of HIV [18]. This might also explain another of our findings, that being an MSM was associated with better QoL. In the qualitative part of the study, MSM participants described greater sense of belonging to a community of people living with HIV [18].

Being in a partnership was also found to be a strong predictor of QoL for total score, as well as several domains (social relations, psychological, level of independence, and environment). Grov and colleagues [15] identified loneliness as an important factor in terms of depressive feelings in older people living with HIV, and not being in a partnership may similarly contribute to lower QoL associated with isolation.

Not having grandchildren was associated with better QoL in some domains. Although our qualitative data indicate that having grandchildren was a source of comfort for some [18]. The quantitative data, however, suggest that being free from the demands of looking after grandchildren was a positive factor with respect to QoL.

Findings in relation to ethnicity are more complex. Multivariate analysis, after adjusting for being on benefits, showed that Black Ethnicity was associated with significantly better QoL in several domains, and when adjusted for the effect of being on benefits, being in the White ethnic group appeared to be a risk factor for low QoL rather than protective against it. More research with a larger sample size is needed to clarify the relationship between ethnicity and QoL.

There were some limitations to our investigation. Our sample was largely London-based, and included few participants from rural environments who might have experienced greater levels of isolation and thus lower quality of life. Also under-represented were non-White and non-Black ethnic groups (e.g. South Asians), and people of Black ethnicity who were not from African countries (e.g. Black Caribbean). Finally, our participants were recruited through HIV clinics and community organizations, thus excluding potential participants who were not engaged with services and support groups, and perhaps experiencing lower quality of life. Further research should aim at expanding the sample of participants, so as to strengthen and widen its findings.

Studies comparing people living with HIV with the general population have shown the former group to have worse quality of life [32], but we are not aware of research comparing PLWH with people with other chronic diseases. As HIV becomes a more manageable condition as a result of new therapies and more people are ageing with HIV, the question of similarities and differences with other chronic disorders is becoming an important one, but complicated by the continued stigmatisation of the virus and of those living with it, and by the increased likelihood that people ageing with HIV will also be living with other chronic disorders, and thus comparison populations should be included in further research.

## Conclusions

Despite growing research into the psycho-social dimensions of ageing with HIV, our understanding of QoL among people living and ageing with HIV remains limited. Our analysis shows that while, overall, older people with HIV reported high levels of QoL and low levels of anxious or depressive feelings, specific variables were associated with QoL, suggesting areas of further intervention and research.

Although, in our sample, ethnicity in relation to QoL did not show clear-cut results, suggesting a complex association needing further research, other associations were more easily identifiable. Unfavourable economic factors were associated with lower QoL, and further research is required to understand the impact of financial concerns linked to ageing in older people living with HIV (e.g. changes to disability benefits). Gender was shown to be a predictor of QoL, with men reporting higher levels. The mechanisms explaining this finding are likely to include differential levels of stigma and feelings of isolation in heterosexual women with HIV, and further research is needed to elucidate the relevant factors. Finally, being in a partnership had a positive effect on QoL, and, surprisingly, not having grandchildren appeared to have a positive impact on some domains of QoL, another finding that requires further investigation, especially given the ageing of the population of people with HIV, and the increased proportion of heterosexuals with children within this population.

These findings, while requiring further exploration, suggest specific policy responses on the part of service providers and policy makers working with this population. We have listed elsewhere several recommendations for policy and practice (see Rosenfeld et al. [18]), but of these, the following are the most relevant given the findings reported here.

First, service providers should be aware of the low QoL and high levels of depressive and anxious feelings among specific groups in older people with HIV (such as those on low income or state benefits, women, those without partners, and those reporting memory concerns), and to be prepared to provide them with the appropriate help and support. Second, older people with HIV should be screened for depressive or anxious feelings, neurocognitive function, and QoL, as part of the regular monitoring of their health. Finally, service providers caring for older people with HIV should not assume that the medical care they receive is necessarily sufficient to identify or manage the psychological and social stressors that can lower their QoL.

## References

[1] May M, Gompels M, Sabin C (2012). Life expectancy of HIV-1-positive individuals approaches normal conditional on response to antiretroviral therapy: UK Collaborative HIV Cohort Study. J Int AIDS Soc.

[2] Nakagawa F, May M, Phillips A (2013). Life expectancy living with HIV: recent estimates and future implications. Curr Opin Infect Dis.

[3] Cresswell F, Fisher M (2013). HIV and the ageing patient. Medicine.

[4] Yin Z, Brown A, Hughes G, Nardone A, Gill O, Delpech V (2014). HIV in the United Kingdom 2014 report: data to end 2013.

[5] Owen G, Catalan J (2012). ‘We never expected this to happen’: narratives of ageing with HIV among gay men living in London, UK. Cult Health Sex.

[6] Ribeiro Nobre N, Kylma J, Kirsi T (2012). I Live Quite a Good Balanced Life: a pilot study on the life experiences of ageing individuals living with HIV. Nurs Res Prac.

[7] Emlet CA (2006). You’re awfully old to have this disease: experiences of stigma and ageism in adults 50 years and older living with HIV/AIDS. Gerontologist.

[8] Emlet CA, Brennan DJ, Brennenstuhl S, Rueda S, Hart TA, Rourke SB (2015). The impact of HIV-related stigma on older and younger adults living with HIV disease: does age matter?. AIDS Care.

[9] Hudson A, Mbewe R, Delpech V. Stigma index study: a continued issue in the UK. In: British HIV association (BHIVA) autumn conference 12–13 November 2015, London; 2015.

[10] Grodensky CA, Golin CE, Jones C, Mamo M, Dennis AC, Abernethy MG (2015). I Should Know Better: the roles of relationships, spirituality, disclosure, stigma, and shame for older women living with HIV seeking support in the south. J Assoc Nurses AIDS Care.

[11] Power L, Bell M, Freemantle I (2010). A national study of people over 50 living with HIV.

[12] Rosenfeld D, Ridge D, Catalan J, Delpech V (2016). Age and life course location as interpretive resources for decisions regarding disclosure of HIV to parents and children: findings from the HIV and later life study. J Aging Stud.

[13] Rosenfeld D, Ridge D, Von Lob G (2014). Vital scientific puzzle or lived uncertainty? Professional and lived approaches to the uncertainties of ageing with HIV. Health Sociol Rev.

[14] Chesney MA, Chambers DB, Taylor JM, Johnson LM (2003). Social support, distress, and well-being in older men living with HIV infection. J Acquir Immune Defic Syndr.

[15] Grov C, Golub SA, Parsons JT, Brennan M, Karpiak SE (2010). Loneliness and HIV-related stigma explain depression among older HIV-positive adults. AIDS Care.

[16] Lyons A, Pitts M, Grierson J, Thorpe R, Power J (2010). Ageing with HIV: health and psychosocial well-being of older gay men. AIDS Care.

[17] Sankar A, Nevedal A, Neufeld S, Berry R, Luborsky M (2011). What do we know about older adults and HIV? A review of social and behavioral literature. AIDS Care.

[18] Rosenfeld D, Anderson J, Ridge D, Asboe D, Catalan J, Collins S (2015). Social support, mental health, and quality of life among older people living with HIV: findings from the HIV and Later Life (HALL) project.

[19] WHO QoL HIV Group (2004). WHOQOL-HIV for quality of life assessment among people living with HIV and AIDS: results from the field test. AIDS Care.

[20] McGowan J, Sherr L, Rodger A, Fisher M, Miners A, Johnson M (2014). Effects of age on symptom burden, mental health and quality of life amongst people with HIV in the UK. J Int AIDS Soc.

[21] Monteiro F, Canavarro MC, Pereira M (2016). Factors associated with quality of life in middle-aged and older patients living with HIV. AIDS Care.

[22] Nokes KM, Coleman CL, Hamilton MJ, Corless IB, Sefcik E, Kirksey KM (2011). Age-related effects on symptom status and health-related quality of life in persons with HIV/AIDS. Appl Nurs Res.

[23] Skevington SM (2012). Is quality of life poorer for older adults with HIV/AIDS? International evidence using the WHOQOL-HIV. AIDS Care.

[24] Deeks SG, Phillips AN (2009). Clinical review: HIV infection, antiretroviral treatment, ageing, and non-AIDS related morbidity. BMJ.

[25] Malaspina L, Woods SP, Moore DJ, Depp C, Letendre SL, Jeste D (2011). Successful cognitive aging in persons living with HIV infection. J Neurovirol.

[26] Valcour V (2013). HIV, aging, and cognition: emerging issues. Top Antivir Med.

[27] Heikinheimo T, Poutiainen E, Salonen O, Elovaara I, Ristola M (2015). Three-decade neurological and neurocognitive follow-up of HIV-1-infected patients on best-available antiretroviral therapy in Finland. BMJ Open.

[28] O’Connell KA, Skevington SM (2012). An international quality of life instrument to assess wellbeing in adults who are HIV-positive: a short form of the WHOQOL-HIV (31 items). AIDS Behav.

[29] Royle J, Lincoln NB (2008). The Everyday Memory Questionnaire–revised: development of a 13-item scale. Disabil Rehabil.

[30] Bolton JE, Breen AC (1999). The Bournemouth Questionnaire: a short-form comprehensive outcome measure. I. Psychometric properties in back pain patients. J Manip Physiol Ther.

[31] Barber T, Bansi L, Pozniak A, Asboe D, Nelson M, Moyle G (2016). Low levels of neurocognitive impairment detected in screening HIV-infected men who have sex with men: the MSM Neurocog Study. Int J STD AIDS.

[32] Miners A, Phillips A, Kreif N, Rodger A, Speakman A, Fisher M (2014). Health-related quality-of-life of people with HIV in the era of combination antiretroviral treatment: a cross-sectional comparison with the general population. Lancet HIV.

